# Construction and Validation of a Novel Ferroptosis-Related Prognostic Model for Acute Myeloid Leukemia

**DOI:** 10.3389/fgene.2021.708699

**Published:** 2022-01-17

**Authors:** Ying Song, Shufang Tian, Ping Zhang, Nan Zhang, Yan Shen, Jianchuan Deng

**Affiliations:** ^1^ Department of Hematology, The Second Affiliated Hospital of Chongqing Medical University, Chongqing, China; ^2^ Hematology Laboratory, The Second Affiliated Hospital of Chongqing Medical University, Chongqing, China

**Keywords:** AML, prognostic model, ferroptosis, survival, prognosis, bioinformatics

## Abstract

Acute myeloid leukemia (AML) is a clonal malignant proliferative blood disorder with a poor prognosis. Ferroptosis, a novel form of programmed cell death, holds great promise for oncology treatment, and has been demonstrated to interfere with the development of various diseases. A range of genes are involved in regulating ferroptosis and can serve as markers of it. Nevertheless, the prognostic significance of these genes in AML remains poorly understood. Transcriptomic and clinical data for AML patients were acquired from The Cancer Genome Atlas (TCGA) and the Gene Expression Omnibus (GEO). Univariate Cox analysis was performed to identify ferroptosis-related genes with prognostic value, and the least absolute shrinkage and selection operator (LASSO) algorithm and stepwise multivariate Cox regression analysis were utilized to optimize gene selection from the TCGA cohort (132 samples) for model construction. Tumor samples from the GEO database (136 samples and 104 samples) were used as validation groups to estimate the predictive performance of the risk model. Finally, an eight-gene prognostic signature (including *CHAC1*, *CISD1*, *DPP4*, *GPX4*, *AIFM2*, *SQLE*, *PGD,* and *ACSF2*) was identified for the prediction of survival probability and was used to stratify AML patients into high- and low-risk groups. Survival analysis illustrated significantly prolonged overall survival and lower mortality in the low-risk group. The area under the receiver operating characteristic curve demonstrated good results for the training set (1-year: 0.846, 2-years: 0.826, and 3-years: 0.837), which verified the accuracy of the model for predicting patient survival. Independent prognostic analysis indicated that the model could be used as a prognostic factor (*p* ≤ 0.001). Functional enrichment analyses revealed underlying mechanisms and notable differences in the immune status of the two risk groups. In brief, we conducted and validated a novel ferroptosis-related prognostic model for outcome prediction and risk stratification in AML, with great potential to guide individualized treatment strategies in the future.

## Introduction

Acute myeloid leukemia (AML) originates from the malignant clonal proliferation of myeloid progenitor cells in bone marrow, peripheral blood, and other tissues and is a highly heterogeneous clinical syndrome. It is the most common type of leukemia in adults, accounting for 2.5% of new cancer cases and 3.1% of new deaths worldwide in 2020, ranking among the top 10 causes of cancer-related deaths (Sung et al., 2021). Currently, chemotherapy and stem-cell transplants remain the primary therapeutic approaches to treat AML patients. Over the past decades, advances in our understanding of AML pathogenesis, combined with the development of intensive consolidation chemotherapy regimens, improved stem-cell transplantation procedures, and enhanced supportive care (Coombs et al., 2016), have contributed to increased overall survival (OS) among AML patients; however, overall outcomes remain unsatisfactory. Accordingly, it is still urgent to explore effective treatment strategies to improve the prognosis of AML patients.

Ferroptosis, first named in 2012, is an iron-dependent regulated cell death characterized by an imbalance in redox homeostasis caused by lipid peroxidation or decreased antioxidant capacity. It can be distinguished from other cell death pathways, including apoptosis, necrosis, and autophagy, through distinct morphologic, biochemical, and genetic characteristics. Glutathione peroxidases (GPXs) are induced through ferroptosis pathways, either directly or indirectly, leading to an overall reduction in the cellular antioxidant capacity, and the formation of excess reactive oxygen species (ROS) (Hirschhorn and Stockwell, 2019). Ferroptosis is primarily regulated by iron metabolism, lipid metabolism, the glutathione (GSH)/glutathione peroxidase 4 (*GPX4*) pathway [such as system Xc- (cysteine-glutamate transporter receptor), the sulfur transfer pathway, the mevalonate (MVA) pathway, the glutamine pathway, and the *p53* regulatory axis], and other pathways, mediated by the regulation of multiple genes (Li J. et al., 2020). Recent accumulating evidence has indicated that ferroptosis participates in the development and progression of various diseases, including cancers, tissue reperfusion injuries, neurodegenerative diseases and so on. And its potent ability to suppress tumor growth and enhance chemotherapeutic sensitivity makes ferroptosis a promising strategy for cancer therapy (Lu et al., 2017).

Only a few studies have explored the relationship between ferroptosis and AML. An *in vitro* experiment demonstrated the ferroptosis inducer erastin induces cell growth inhibition and alters the resistance of AML cells against chemotherapeutic agents (cytarabine and doxorubicin) through ferroptosis and necroptosis (Yu et al., 2015). As an anti-malaria drug, dihydroartemisinin (DHA) has been shown to specifically inhibit the growth of AML cells but shared no toxicity to normal hematopoietic progenitor cells, which is mediated by the autophagy-dependent degradation of ferritin (Du et al., 2019). Glutathione peroxidase-1 (*GPX1*), closely bound up with the process of ferroptosis, *TP53* regulation, ROS metabolism, and GSH metabolism, was confirmed to be highly expressed in AML and associated with poor prognosis of AML (Wei R. et al., 2020). Nevertheless, there is not yet a comprehensive description of how ferroptosis functions in AML. Consequently, we aimed to explore the prognostic value of ferroptosis-associated genes, reveal the underlying mechanisms, and construct a novel prognosis prediction signature for AML according to the expression levels of ferroptosis-related genes.

In the current research, RNA expression profiles and complete clinical information for AML patients were obtained from The Cancer Genome Atlas (TCGA) and the Gene Expression Omnibus (GEO). Then we conducted an eight-gene prognostic prediction model based on identified ferroptosis-related genes with prognostic value, which was validated in two GEO cohorts through survival analysis, independent prognostic analysis, and receiver operating characteristic (ROC) curve analysis. Finally, Gene Ontology (GO), Kyoto Encyclopedia of Genes and Genomes (KEGG) analyses and single-sample gene set enrichment analysis (ssGSEA) were performed to seek for several underlying mechanisms.

## Materials and Methods

### Data Collection and Processing

The Cancer Genome Atlas (TCGA, https://portal.gdc.cancer.gov/) is a comprehensive database sponsored by the government of the United States, collecting more than 11,000 cases across 33 tumor types (Hutter and Zenklusen, 2018). The Gene Expression Omnibus (GEO, https://www.ncbi.nlm.nih.gov/geo/) is the largest public resource for gene expression data, storing chips, second-generation sequencing, and other high-throughput sequencing data (Barrett et al., 2013). The mRNA expression profiles and clinical information of AML bone marrow samples were downloaded from TCGA (Weinstein et al., 2013). Two gene expression matrices (GSE37642_series_matrix, GSE71014_series_matrix) regarding AML studies and the corresponding probe annotation platform files (GPL570, GPL96) were acquired from the GEO database (Li et al., 2013; Chuang et al., 2015). The standard for screening samples was as follows: 1) the samples were derived from the bone marrow of patients who were diagnosed with AML, based on relevant diagnostic standards; 2) the samples included integrated survival data; 3) the survival time of the patients was longer than 0 day. Because all data used in this study were gathered from public databases, no ethics committee approvals were required. A total of 60 ferroptosis-related genes were obtained by searching the previous literature (Stockwell et al., 2017; Doll et al., 2019a; Bersuker et al., 2019; Hassannia et al., 2019) (Supplementary Table S1).

The Ensembl identifies in the gene matrix downloaded from the TCGA database were converted into gene symbols by referring to the information from GENCODE (version 32, www.gencodegenes.org/). Probe set identifiers were converted into gene symbols according to the corresponding annotation documents from the GEO website. For multiple probe sets associated with a unique gene symbol, the average value of gene expression was used. To minimize the impacts on subsequent analyses as to the greatest extent possible, all genes with no expression detected in any AML samples were removed. We then extracted and intersected the ferroptosis-related genes from the three cohorts and corrected for batch effects using the sva R package (Leek et al., 2012) for the convenience of comparisons.

### Prognostic Signature Construction and Validation

After combining the expression levels of ferroptosis-related genes with survival time and survival state, univariate Cox regression analysis was conducted in 132 AML samples from the TCGA database to screen survival-associated genes, and finally 20 genes were obtained with *p* < 0.05 as the cutoff value. Thereafter, a least absolute shrinkage and selector operation (LASSO) algorithm (Tibshirani, 1997) was performed to fit the model 1,000 times while adjusting the gene screening and complexity to obtain better performance parameters and to avoid the appearance of overfitting using the glmnet R package. A novel eight-gene prognostic signature for AML emerged following stepwise multivariate Cox regression analysis. The expression levels of these eight genes between AML patients and corresponding normal samples were compared by GEPIA (Gene Expression Profiling Interactive Analysis, http://gepia.cancer-pku.cn/), an online tumor data mining server based on the TCGA and GTEx projects (Tang et al., 2017). The threshold values for |log_2_FC| and *p*-value were 1 and 0.01, respectively.

In the light of the established risk scoring formula, the risk scores of each patient in the TCGA and GEO databases were calculated and grouped into high- or low-risk groups by the median value of the risk score in the training set. Survival curves, risk curves, and ROC curves for the three cohorts were visualized separately with the survival R package, survminer R package, heatmap R package, and timeROC R package. Independent prognostic analyses were conducted and presented as forest maps for the TCGA-LAML and GSEE37642-GPL570 cohorts to determine whether the model was worthy of being a prognostic factor independent of other clinical traits.

### Functional Enrichment Analysis

We carried out functional enrichment analyses to identify relevant signaling pathways and reveal the underlying molecular mechanisms associated with the biological processes. First, risk difference analysis was operated by using the limma package and Wilcox test to screen the differentially expressed genes (DEGs) between the high-risk and low-risk groups, using false-discovery rate (FDR) < 0.05 and | log fold change (FC) | > 1 as the significance level. Afterwards, GO and KEGG analyses were performed with the clusterProfiler R package (Yu et al., 2012), based on the DEGs, filtered by *p* < 0.05. The top 10 GO analysis results for biological processes (BP), cellular components (CC), and molecular function (MF) terms and the top 30 results from the KEGG analysis are displayed as bar plots and bubble plots, respectively. According to the annotated gene set (Supplementary Table S2), ssGSEA analysis was performed to get scores for immune cells and immune-related functions for each sample in the TCGA-LAML cohort with the GSVA R package (Hänzelmann et al., 2013). Differences between the two risk groups were analyzed and visualized as box plots using the limma R package, ggpubr R package, and reshape2 R package. The threshold value for significance was *p* < 0.05.

### Statistical Analysis

Statistical analyses were performed using R software (version 3.6.1, https://www.r-project.org/) and Perl software (https://www.perl.org/). The mean value, range, and proportions of clinical information in the three sequences were analyzed by SPSS version 24. The Wilcox test was used to identify the DEGs between the two risk groups. Generally, *p* < 0.05 was considered significant unless otherwise specified.

## Results

### Patient Characteristics

As noted above, gene expression data and clinical information for AML patients were acquired from the public databases TCGA and GEO. After filtering, as described in the Methods, 132 AML samples from the TCGA-LAML project were regarded as the training group, whereas 136 samples from the GSE37642-GPL570 matrix, and 104 samples from the GSE71014 matrix were treated as the validation group. The clinical information of TCGA was relatively abundant, including age, gender, race, ethnicity, cytogenetics risk category, platelet count, French–American–British (FAB) category, hemoglobin value, white blood cell (WBC) value, blast cell percentages in peripheral blood and bone marrow, and cytogenetic abnormality. The clinical data included in GSE37642-GPL570 were limited to age, FAB category, *runx1*-*runx1t1* fusion status, and *runx1* mutation status. No clinical information was provided in GSE71014; therefore, the independent prognostic analysis was based on the TCGA-LAML and GSE37642-GPL570 projects. The average ages of the TCGA-LAML and GSE37642-GPL570 cohort were 53 years (range: 21–88 years) and 56 years (range: 18–85 years), respectively. M1 and M2 were the most common AML types in the two cohorts. Detailed clinical characteristics for the three cohorts are listed in Table 1.

**TABLE 1:** Clinical characteristics of AML patients in the three cohorts.

Variable	TCGA-LAML cohort (*n* = 132)	GSE37642-GPL570 cohort (*n* = 136)	GSE71014 cohort (*n* = 104)
Average age (year)	53.27 (21–88)	55.51 (18–85)	
Gender			
Female	61 (46.2%)		
Male	71 (53.8%)		
Lab data			
Platelet (10^9^/L)	65.12 (9–351)		
Hemoglobin (g/dl)	9.53 (6–13)		
WBC (10^9^/L)	34.38 (1–224)		
Peripheral blasts (%)	65.67 (0–100)		
Bone marrow blasts (%)	38.11 (0–97)		
Cytogenetics risk			
Favorable	30 (22.7%)		
Intermediate/Normal	73 (55.3%)		
Poor	27 (20.5%)		
Unknown	2 (1.5%)		
Vital status			
Alive	52 (39.4%)	38 (27.9%)	68 (65.4%)
Dead	80 (60.6%)	98 (72.1%)	36 (34.6%)
FAB			
M0	12 (9.1%)	8 (6.0%)	
M1	32 (24.2%)	29 (21.3%)	
M2	32 (24.2%)	47 (34.6%)	
M3	14 (10.6%)	7 (5.1%)	
M4	27 (20.5%)	17 (12.5%)	
M5	12 (9.1%)	19 (14.0%)	
M6	2 (1.5%)	7 (5.1%)	
M7	1 (0.8%)	1 (0.7%)	
Unknown	0 (0.0%)	1 (0.7%)	
*Runx1*-*runx1t1* fusion			
Yes		7 (5.1%)	
No		129 (94.9%)	
*Runx1* mutation			
Yes		16 (11.8%)	
No		108 (79.4%)	
Unknown		12 (8.8%)	

TCGA, The Cancer Genome Atlas; AML, acute myeloid leukemia; Lab, laboratory; WBC, white blood cell; FAB, French-American-British.

### Construction and Validation of the Prognostic Model

**FIGURE 1:**
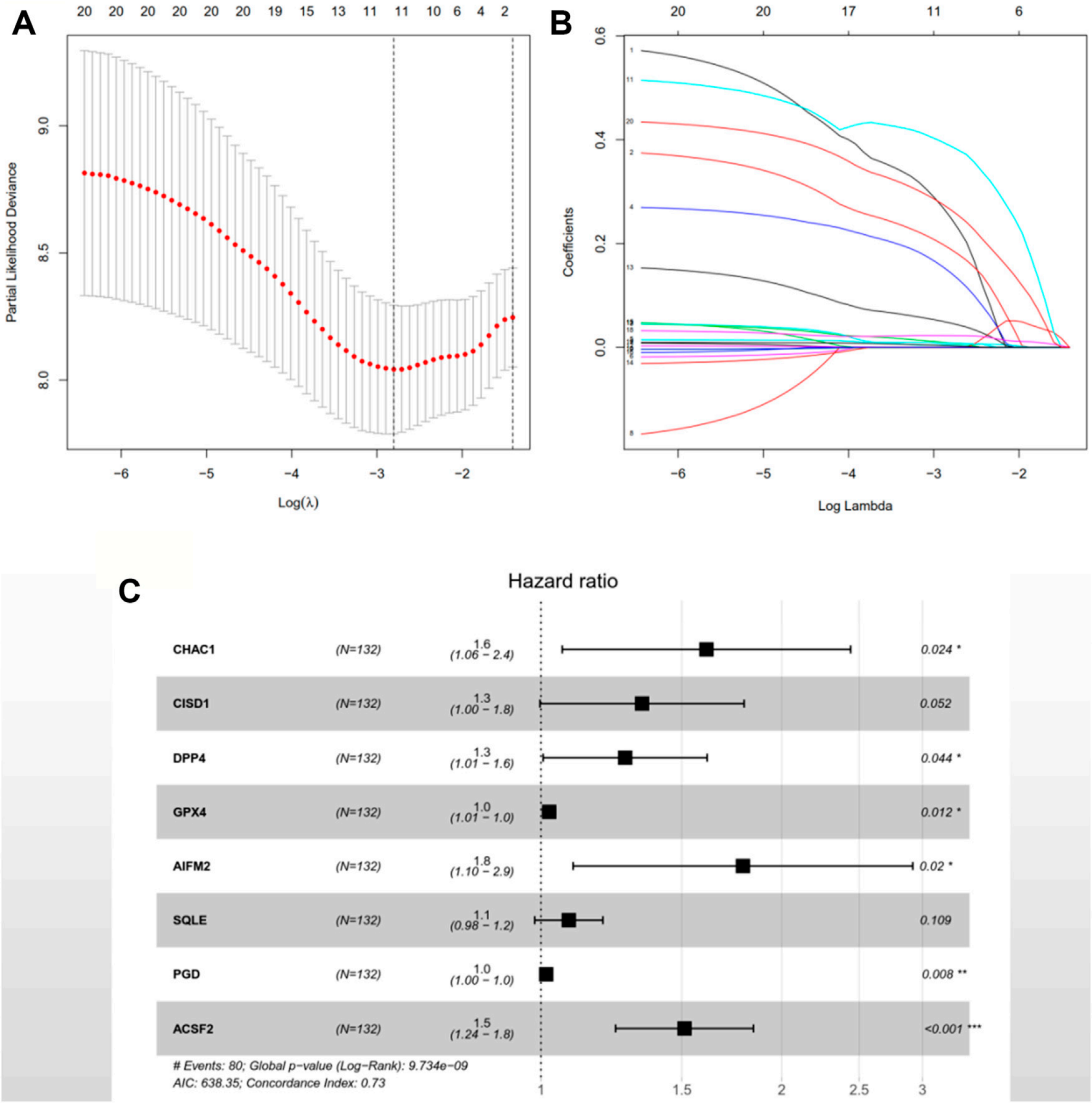
An eight-gene prognostic signature was constructed based on the LASSO algorithm in the training group TCGA-LAML. **(A)** 1,000-fold cross-validation was performed to choose the lambda value with the minimum cross-validation error. **(B)** The coefficients of model genes were calculated to remove highly correlated genes. **(C)** A forest map of the eight featured genes. HR > 1 indicates the gene is a high-risk gene; otherwise, the gene is a low-risk factor. *p* < 0.05 illustrates the independence of the gene for predicting the prognosis of AML patients, and vice versa. TCGA: The Cancer Genome Atlas; AML: acute myeloid leukemia; LASSO: least absolute shrinkage and selection operator; HR: hazard ratio.

After the previous data processing, 56 intersecting genes were used for univariate Cox analysis in the training group, obtaining 20 ferroptosis-related genes with survival significance by *p* < 0.05 (Table 2). The LASSO algorithm and stepwise multivariate Cox regression analysis were applied, which resulted in the generation of an eight-gene prognostic model (Figure 1). The patient’s risk score = 0.476551252 × *CHAC1* + 0.291108125 × *CISD1* + 0.242536399 × *DPP4* + 0.02380646 × *GPX4 +* 0.581670513 × *AIFM2 +* 0.080298868 × *SQLE* + 0.01518642 × *PGD* + 0.413485022 × *ACSF2* (Table 3). Among them, the expression of *CHAC1*, *CISD1*, *GPX4*, *AIFM2*, *SQLE*, and *PGD* were lower in AML than in normal tissues, while *DPP4* and *ACSF2* had a higher expression (Supplementary Figure S1). Since the calculated hazard ratio (HR) for each of these eight genes was greater than 1, they were judged as being high-risk genes that may be negatively associated with the survival of AML patients, and six of them (*CHAC1*, *DPP4*, *GPX4*, *AIFM2*, *PGD*, and *ACSF2*) were identified as independent prognostic genes with *p* < 0.05.

**TABLE 2:** Ferroptosis-related genes with survival significance in the univariate Cox regression analysis of the TCGA-LAML cohort.

Gene name	HR	HR.95L	HR.95H	*p*-value
G6PD	1.042662659	1.021730497	1.064023657	5.40E-05
GPX4	1.029261441	1.01470048	1.044031353	7.26E-05
LPCAT3	1.26784648	1.126151189	1.427370244	8.68E-05
AIFM2	2.35902572	1.522954065	3.654084172	0.000121029
ACSF2	1.416086083	1.183993793	1.693674245	0.000139401
SLC1A5	1.034660853	1.007310121	1.062754218	0.012673093
GSS	1.084599685	1.017485242	1.15614107	0.012708482
HSPB1	1.021101123	1.004464707	1.038013079	0.012721046
FTH1	1.004820236	1.001006148	1.008648857	0.013203117
FADS2	1.044215109	1.008006196	1.081724694	0.01626862
DPP4	1.315339205	1.044859818	1.655836691	0.01961824
KEAP1	1.042701327	1.006702842	1.079987074	0.019667367
PGD	1.011288392	1.001788789	1.020878077	0.019747746
CS	1.073417219	1.010825563	1.139884634	0.020820444
GOT1	1.182375661	1.02398682	1.365263865	0.022430936
SQLE	1.106383386	1.013803784	1.207417269	0.023363045
CHAC1	1.533037962	1.053292623	2.231293889	0.025674875
CISD1	1.33620604	1.026729735	1.738964521	0.031067487
RPL8	1.00324892	1.000260989	1.006245776	0.033052858
NCOA4	0.992630046	0.98539641	0.999916784	0.047449628

TCGA, The Cancer Genome Atlas; AML, acute myeloid leukemia; HR, hazard ratio.

**TABLE 3:** Multivariate Cox regression analysis of model genes in the TCGA-LAML cohort.

Gene name	Coef	HR	HR.95L	HR.95H	*p*-value
CHAC1	0.476551252	1.610510569	1.063220885	2.439515936	0.024492634
CISD1	0.291108125	1.337909237	0.997173742	1.79507447	0.052247121
DPP4	0.242536399	1.274477638	1.006482968	1.613830837	0.044050084
GPX4	0.02380646	1.024092096	1.00530429	1.043231021	0.011737265
AIFM2	0.581670513	1.789024525	1.096982582	2.917647739	0.01975936
SQLE	0.080298868	1.083610876	0.982343805	1.195317287	0.108691658
PGD	0.01518642	1.015302319	1.004037904	1.026693112	0.00763266
ACSF2	0.413485022	1.51207824	1.239707104	1.844290959	4.50E−05

TCGA, The Cancer Genome Atlas; AML, acute myeloid leukemia; HR, hazard ratio; Coef, coefficient.

**FIGURE 2:**
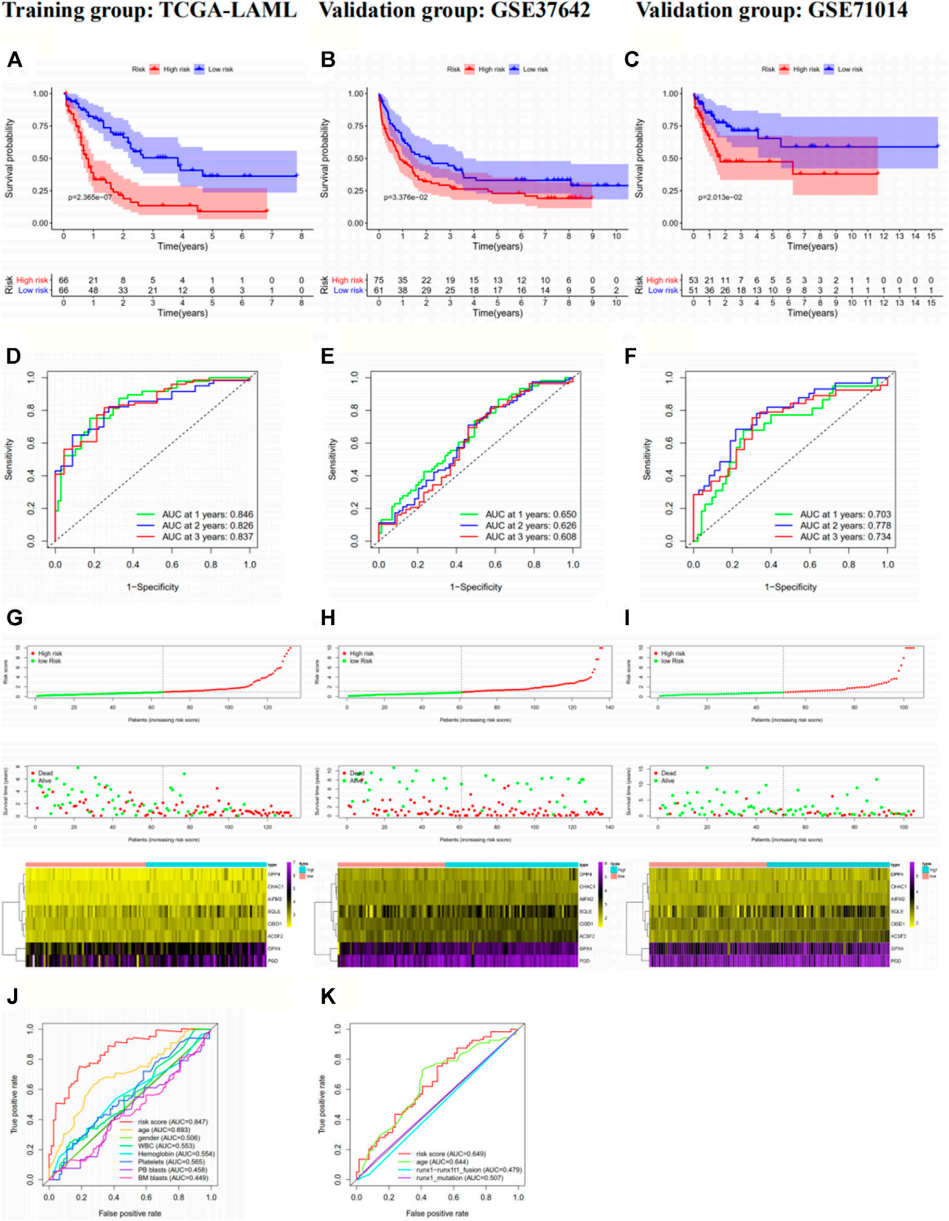
Prognostic analysis of the eight-gene signature in the training group TCGA-LAML **(A, D, G,** and **J)**, validation group GSE37642-GPL570 **(B, E, H,** and **K)** and validation group GSE71014 **(C, F,** and **I)**. In the survival curve **(A–C)**, the number of surviving patients decreased over time. *p* < 0.05 indicates significant differences in survival between the two groups. In the risk score curve (**G-I**), the top shows the risk score for each patient; green dots represent low-risk patients, and red dots represent high-risk patients. The middle shows the distribution of survival status and survival times for each patient, with red dots representing dead patients and green dots representing living patients. The number of deaths increased with increasing risk scores. The bottom is the expression heat map of the eight model genes, with the left showing the low-risk group and the right showing the high-risk group. The gene expression levels largely increase with the risk scores. In the ROC curves, we used the risk score as the only feature for 1-, 2-, and 3-years OS **(D–F)**. In addition, we compared the 1-year OS prediction accuracy of the risk score with other single clinical characteristics **(J–K)**. TCGA, The Cancer Genome Atlas; AML, acute myeloid leukemia; ROC, receiver operating characteristic; OS, overall survival.

**FIGURE 3:**
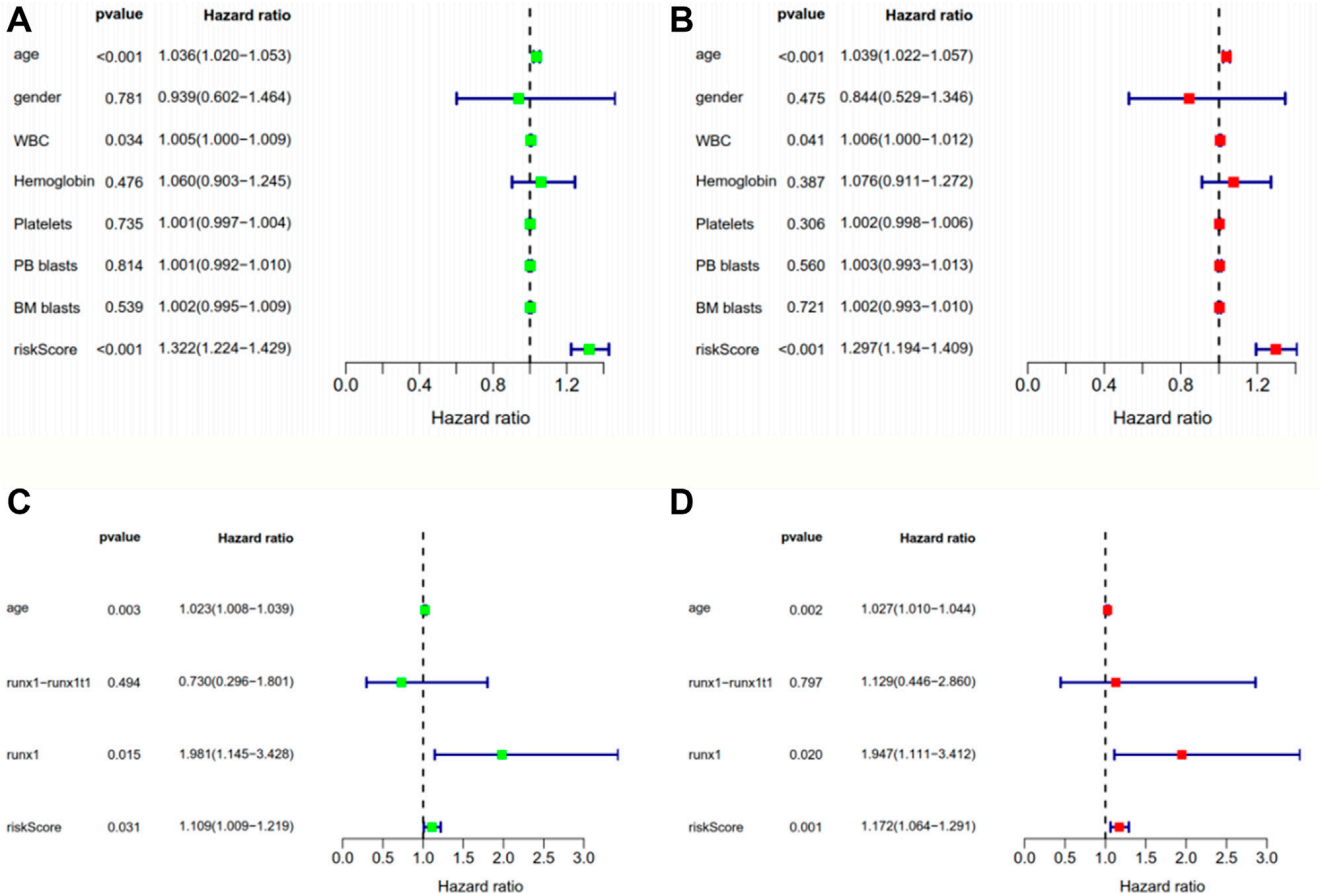
Univariate **(A, C)** and multivariate **(B, D)** Cox regression analyses of clinical information and risk scores in the training group TCGA-LAML **(A, B)** and the validation group GSE37642-GPL570**(C, D)**. *p* < 0.05 indicates that a factor is associated with the overall survival of AML. HR > 1 indicates the factor is a high-risk factor and is negatively correlated with the prognosis of AML patients. The risk score obtained from the model was found to be an independent prognostic factor, with *p* < 0.05 in both the univariate and multivariate analyses. TCGA, The Cancer Genome Atlas; AML, acute myeloid leukemia; HR, hazard ratio.

The risk scores of the AML patients in the training and validation groups were calculated based on the above formula and differentiated into high- and low-risk groups for subsequent analyses (Supplementary Table S3). The survival analyses (Figures 2A–C) between the two groups in each dataset demonstrated significant differences (training group TCGA-LAML: *p* = 2.365e−07; validation group GSE37642-GPL570: *p* = 3.376e−02; validation group GSE71014: *p* = 2.013e−02), especially in the training set. Over time, there was an intuitive advantage in the low-risk group of higher OS and lower mortality compared with the high-risk group. The risk score curves (Figures 2G–I) successfully separated the high-risk group from the low-risk group, and the patients’ risk scores were linked from left to right. The survival state diagrams (Figures 2G–I) clearly show the survival state of each patient, and the number of deaths increased with the risk score. The heat maps (Figures 2G–I) revealed the gene expression values of the model genes in each sample by color distribution. Generally speaking, the expression level of each gene increased with the risk score, which further indicated that the model genes were all high-risk genes. To our great satisfaction, the area under the ROC curve (AUC) values (Figures 2D–F) for the training set were relatively high (1-year: 0.846; 2-years: 0.826; 3-years: 0.837), verifying the great predictive performance of the signature. By contrast, the results were barely acceptable in the validation groups, which might be due to differences in the feature distribution among the datasets. Furthermore, another ROC curve (Figure 2J–K), which combined the risk score and the detailed clinical information, was visualized for the TCGA-LAML and GSE37642-GPL570 datasets, indicating the advantage of the established model for prognosis prediction compared with single clinical information. Finally, univariate and multivariate analyses conducted for the TCGA-LAML and GSE37642-GPL570 cohorts declared the independent prognostic ability of the risk score (Figure 3).

### GO, KEGG, and ssGSEA Analysis

**FIGURE 4:**
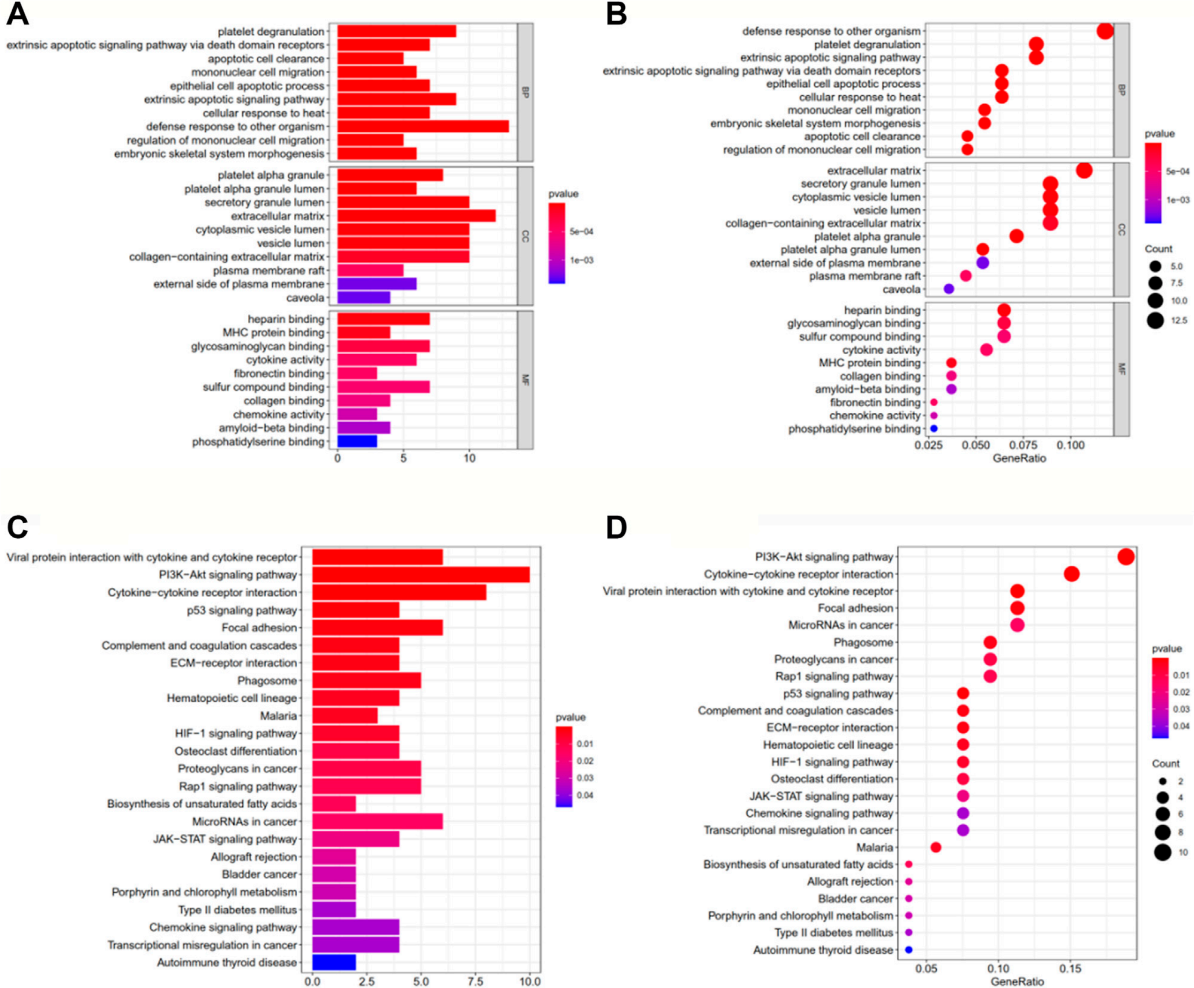
Gene Ontology **(**GO; **A, B)** and Kyoto Encyclopedia of Genes and Genomes **(**KEGG; **C, D)** enrichment analyses of differentially expressed genes in the training set TCGA-LAML. **(A, B)** The top 10 results of the GO enrichment analysis. **(C, D)** are the most significant KEGG pathways. In the bar charts **(A, C)**, the length of each column represents the number of genes enriched, with longer columns indicating more enriched genes. The colors of the columns represent the significance of the enrichment, with redder columns being more significant. In the bubble diagrams **(B, D)**, the sizes of the circles represent the number of genes enriched, with larger circles indicating more genes enriched. The colors of the circles represent the significance of the enrichment, with the redder circles being more significant.

GO and KEGG enrichment analyses were performed on the DEGs identified between the two risk groups in the TCGA-LAML cohort (Supplementary Table S4, S5). Figure 4 shows the top 10 GO terms and top 30 KEGG pathways with the most significance. Most enriched pathways were related to immunity, metabolism, and cancer, indicating the accuracy of our model.

**FIGURE 5:**
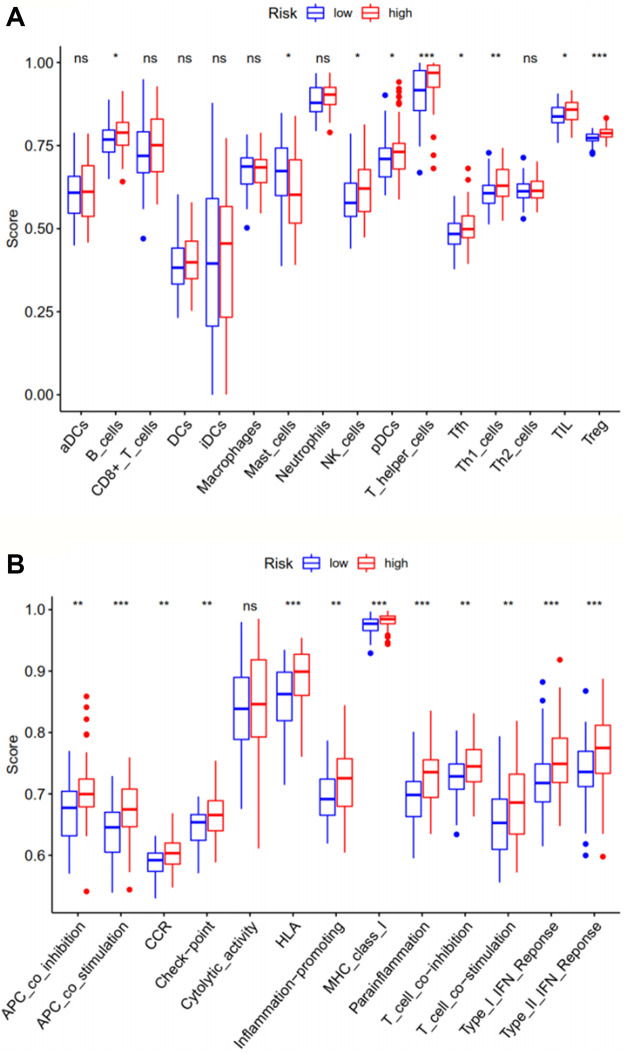
The ssGSEA analysis compares immune cells (**A**) and immune-related functions (**B**) between the two risk groups in the training set TCGA-LAML. ***, *p* < 0.001; **, *p* < 0.01; *, *p* < 0.05; ns, *p* > 0.05. ssGSEA: single-sample gene set enrichment analysis.

The ssGSEA analysis compared immune activity between the high-risk group and the low-risk group in the TCGA-LAML cohort, based on scores for 16 types of immune cells and 13 types of immune functions. The results (Figure 5) indicated significant differences in immune activity between the two groups, especially for aspects of antigen-presenting cell (APC) co-stimulation, human leukocyte antigen (HLA), major histocompatibility complex (MHC) class I, parainflammation, type I interferon (IFN) response, type II IFN response, T helper cells, and T regulatory cells (Tregs), with *p* values <0.001. Immune function essentially differed between the two risk groups, except for cytolytic activity, suggesting these ferroptosis-related genes may have a certain impact on the survival and prognosis of tumor patients through relevant immune pathways, although the exact mechanism requires further exploration.

## Discussion

AML is a clinically and genetically heterogeneous disorder of hematopoietic stem cells, with an annual incidence that is consistently higher than 4.2 per 100,000 population in the United States and a mortality rate of 2.8 per 100,000 population, based on SEER (Surveillance, Epidemiology, and End Results) data (Shallis et al., 2019). With the rapid development of high-throughput sequencing technology, the available treatment options for AML patients are becoming increasingly diverse. In addition to conventional chemotherapy and stem-cell transplantation, a large number of targeted small-molecule drugs have been approved for AML, and which provide certain patients with beneficial outcomes. However, the poor prognosis and high mortality of AML is still a great challenge and undisputed fact.

Cell death, mainly composed of uncontrolled cell death and programmed cell death, represents an irreversible and fundamental biological process that is necessary to maintain the normal functions and morphologies of tissues (D’Arcy, 2019). It is the basis of embryonic development, tissue homeostasis, and immune mechanisms. As a double-edged sword affecting the development and progression of cancers, inhibiting cell death can promote oncogenesis and increase chemotherapeutic resistance. By contrast, the promotion of cell death has been utilized as a direct therapeutic approach to cancers (Strasser and Vaux, 2020). Ferroptosis is a newly characterized form of programmed cell death induced by iron-dependent lipid peroxidation and has been strongly linked to the pathophysiology of various diseases, including cancers, inflammatory diseases, neurological diseases, acute kidney injury, age-related macular degeneration, psoriasis, and hemolytic disorders (Li J. et al., 2020). It has been a hot topic and attracted the most attention of researchers on how to intervene in the occurrence, development and treatment of tumors through regulating pathways activating or inhibiting ferroptosis.

Recently, a large number of studies have highlighted the place of targeted ferroptosis in the treatment of AML. Current approaches to treat AML by modulating iron metabolism can be broadly classified into four categories: iron chelators, modulation of proteins involved in iron metabolism, induction of ferroptosis, and delivery of anti-leukemic drugs by ferritin (Weber et al., 2020). Iron chelators prevent the induction of ferroptosis, and deferoxamine (DFO) exerts anti-leukemic effects by blocking ROS elaboration and iron-dependent enzymes. Targeted peptides and antibodies against transferrin are also used to treat AML, and transferrin has been used to deliver anti-leukemic drugs (Grignano et al., 2020). While APR-246 and ATPR (a novel all-trans retinoic acid derivative) can both induce ferroptosis of AML cells and become targets for AML therapy (Du et al., 2020; Birsen et al., 2021). High mobility group box 1 (HMGB1) is a key regulator of erastin-induced ferroptosis via the RAS-JNK/p38 pathway and is also expected to be a potential drug target in leukemia (Ye et al., 2019). Furthermore, since mitochondria are closely associated with iron metabolism and ROS production, several studies have recently elucidated mitochondria metabolism as a therapeutic strategy for AML, and multiple drugs are undergoing clinical trials (Panina et al., 2021).

At the present research, we extracted the expression levels of ferroptosis-related genes from gene expression sequences and identified genes with prognostic significance by univariate Cox regression analysis. A clinical prognostic model was conducted to predict the survival risk of AML patients by the LASSO algorithm, and the accuracy was validated in two additional independent cohorts. There was an evident prolonged OS time and lower mortality rate of the low-risk group. In addition, the model was determined to be an independent prognostic factor, with high AUC values indicating the accuracy of the risk assessment model. Besides, functional enrichment analyses were carried out to look for possible pathways ferroptosis-related genes may be involved in regulating the biological processes of AML patients.

A novel and effective prognostic signature based on eight ferroptosis-related genes (*CHAC1*, *CISD1*, *DPP4*, *GPX4*, *AIFM2*, *SQLE*, *PGD*, and *ACSF2*) were identified in the TCGA-LAML project and verified in two other independent cohorts from the GEO database. These eight featured genes were all high-risk genes and were negatively correlated with the prognosis of AML patients. Up to now, numerous researchers have devoted themselves to investigating the pathogenic mechanisms of these genes in various cancers and their relationship with prognosis. GSH is a powerful weapon for antioxidation and free radical scavenger found in living organisms, with functions of maintaining redox homeostasis, detoxifying, anti-aging, enhancing immunity, and also one of the important regulatory mechanisms of ferroptosis. Its deficiency weakens the capacity to defend against oxidative stress and promotes tumor progression, whereas elevated GSH levels increase the antioxidant ability and tolerance of tumor cells (Bansal and Simon, 2018). *CHAC1* is involved in the degradation of GSH and is indispensable for the process of ferroptosis. GSH levels are reduced due to cystine-starvation in triple-negative breast cancer cells, suggesting that *CHAC1* acts as an oncogene in triple-negative breast cancer, which is consistent with the findings of our AML study (Chen et al., 2017). *CISD1*, also called *mitoNEET*, is a low-molecular-weight protein localized on the outer membrane of mitochondria, which inhibits ferroptosis by protecting against mitochondrial lipid peroxidation (Yuan et al., 2016). Sohn et al. demonstrated the significance of *mitoNEET* for promoting the proliferation and growth of breast cancer cells through the maintenance of mitochondrial homeostasis, highlighting *mitoNEET* as a promising target for anti-tumor therapy (Sohn et al., 2013). Similarly, the anti-leukemia potential of *mitoNEET* in refractory or recurrent B-cell acute lymphoblastic leukemia has been emphasized by Geldenhuys et al. (2019). *DPP4*, which encodes the T-cell surface antigen CD26, is a serine protease that is overexpressed in the intestine, liver, pancreas, placenta, thymus, and circulating blood. According to the study, the tumor suppressor gene *p53* inhibits ferroptosis by directly suppressing the activity of *DPP4*, suggesting the important role of *DPP4* in the regulation of ferroptosis (Kang et al., 2019). A recent study based on bioinformatics and immunohistochemistry suggested that dysregulation of *DPP4* expression in AML severely affects chemotherapy sensitivity, yet the mechanisms involved are unclear (Wei et al., 2021). As a key regulator of ferroptosis, the inhibition of *GPX4* makes drug-resistant tumor cells susceptible to ferroptosis, yet some tumor cells develop resistance mechanisms independent of ferroptosis (Shin et al., 2018). A study analyzed the role of the GPX family in AML and found high expression of *GPX4* in AML samples was associated with poor overall survival (Wei J. et al., 2020). *AIFM2*, the former name of ferroptosis suppressor protein 1 (*FSP1*), is a DNA-binding oxidoreductase protein derived from the mitochondria that has been reported to cooperate with *GPX4* and GSH to suppress phospholipid peroxidation and ferroptosis (Doll et al., 2019b. Some studies have demonstrated that *AIFM2* is effective for the suppression of tumorigenicity in epithelial ovarian cancer and lung cancer cells (Notaridou et al., 2011; Lu et al., 2016). Cholesterol metabolism plays an important role in the regulation of tumor biological processes, including ferroptosis, and a large number of investigators were devoted to exploring the maximum potential of cholesterol metabolism in tumor-targeted therapy (Xu et al., 2020). As a key rate-limiting enzyme of cholesterol metabolism, *SQLE*, has been closely connected with the proliferation and metastasis of various cancers and has been used to predict poor prognosis, such as in nonalcoholic fatty liver disease-induced hepatocellular carcinoma, nasopharyngeal carcinoma, lung squamous cell carcinoma, and breast cancer (Liu et al., 2018; Ge et al., 2019; Li L. et al., 2020; Kim et al., 2021). *PGD* is an enzyme that mediates the third step of the pentose phosphate pathway (PPP) and is overexpressed in multiple tumor cells, promoting the proliferation, survival, and metastasis of tumor cells through reprogrammed tumor bioenergetics. In addition, its overexpression induces the development of chemotherapy resistance in lung cancer, thyroid cancer, and ovarian cancer (Sarfraz et al., 2020). Moreover, *PGD* is involved in the growth and therapy resistance of AML cell lines (Bhanot et al., 2017). *ACSF2*, encodes regulators of acyl-CoA synthesis, there are few investigations concerning the relationship between *ACSF2*, and the prognosis of AML patients to date. A retrospective study identified *ACSF2* as a sensitive and specific molecular marker of recurrent deep vein thrombosis by gene expression profiling for the first time, which makes great significance for selecting groups requiring extended anticoagulant therapy after confirmed in larger prospective studies (Montes et al., 2016). Yao et al. observed the iron chelator deferoxamine could promote the recovery of the traumatic spinal cord by downregulating *ACSF2*, inferring drugs specifically targeting ferroptosis are expected to be a novel treatment avenue for spinal cord injury (Yao et al., 2019). Although previous studies have confirmed the important role of ferroptosis-related genes in certain diseases, further investigations are needed to explore specific mechanisms associated with the pathogenesis and prognosis of the disease.

As expected, the enriched pathways are primarily associated with immunity, metabolism and cancer. PI3K-AKT signaling pathway was the most significant pathway of enrichment. Activation of the PI3K-AKT-mTOR pathway has been found to protect cancer cells from ferroptosis, while inhibition of the pathway enhances the effect of ferroptosis-induced cancer treatment (Yi et al., 2020). And p53 signaling pathway plays a dual role in the regulation of ferroptosis ADDINADDIN (Liu et al., 2020). These indicate that these pathways may affect tumorigenic progression by inhibiting or inducing ferroptosis. In the ssGSEA analysis, the high-risk group had a higher proportion of regulatory T cells and T helper cells. According to previous studies, regulatory T cells can help leukemic cells achieve escape from immune surveillance to promote tumor progression, which is associated with the poor prognosis of AML patients (Guo et al., 2020). Whereas different types of T helper cells play their respective roles in tumor immunity, and its specific mechanism in leukemia remains largely unknown. Both the type I IFN response and type II IFN response were higher in the high-risk group. Some studies have instructed that IFN can kill tumor cells by inducing ferroptosis (Zitvogel and Kroemer, 2019). Yet some studies have illustrated the cancer-promoting effect of IFN (Castro et al., 2018), a clear understanding of the mechanisms involved needs to be demonstrated through more investigations.

Some limitations must be mentioned in our research. First, our data were obtained from the public TCGA database, which has a higher rate of censored data, and affecting the reliability of the results. The lack of additional clinical information, such as data regarding chemotherapy, transplantation, and other attempted therapeutic methods, the number of response events, and event-free survival (EFS), limited our deep investigations of the model and made our analysis less extensive and comprehensive. Second, the sample sizes of the training and validation sets were far from sufficient to support the widespread application of the developed model in clinical practice, and larger volumes of data from real-world trials are still needed. Third, we did not consider whether the testing performance of the model might be reduced when applied across different AML subtypes.

In summary, although we successfully developed a ferroptosis-related prognostic signature to predict the survival time of patients with AML, further investigations are still needed to elucidate the accuracy and universality of the model.

## Conclusion

In conclusion, according to the expression level of ferroptosis-associated genes in AML, we established an effective survival scoring model by bioinformatics analysis method and verified it as an independent prognostic factor for OS of AML patients. Furthermore, we performed functional analysis to explore the possible pathways and the relationships between ferroptosis and immunity. However, due to the limitations of the study, additional investigations should be done to further explore the significance and mechanisms of ferroptosis in AML.

## Data Availability

The original contributions presented in the study are included in the article/Supplementary Material, further inquiries can be directed to the corresponding authors.

## Abbreviations

AML, acute myeloid leukemia; AUC; area under the curve; CI, confidence interval; DEGs, differentially expressed genes; FAB, French-American-British; GSH; glutathione; GPX, glutathione peroxidase; GEO, Gene Expression Omnibus; GO, Gene Ontology; HR, hazard ratio; KEGG, Kyoto Encyclopedia of Genes and Genomes; LASSO, least absolute shrinkage and selection operator; OS, overall survival; ROS, reactive oxygen species; TCGA, The Cancer Genome Atlas; ROC, receiver operating characteristic; ssGSEA, single-sample gene set enrichment analysis.
