# Supplementary figures and images for: Construction and Validation of a Novel Ferroptosis-Related Prognostic Model for Acute Myeloid Leukemia
Source: Front Genet. 2022 Jan 17;12:708699. doi: 10.3389/fgene.2021.708699 (PMC8803125; doi:10.3389/fgene.2021.708699)

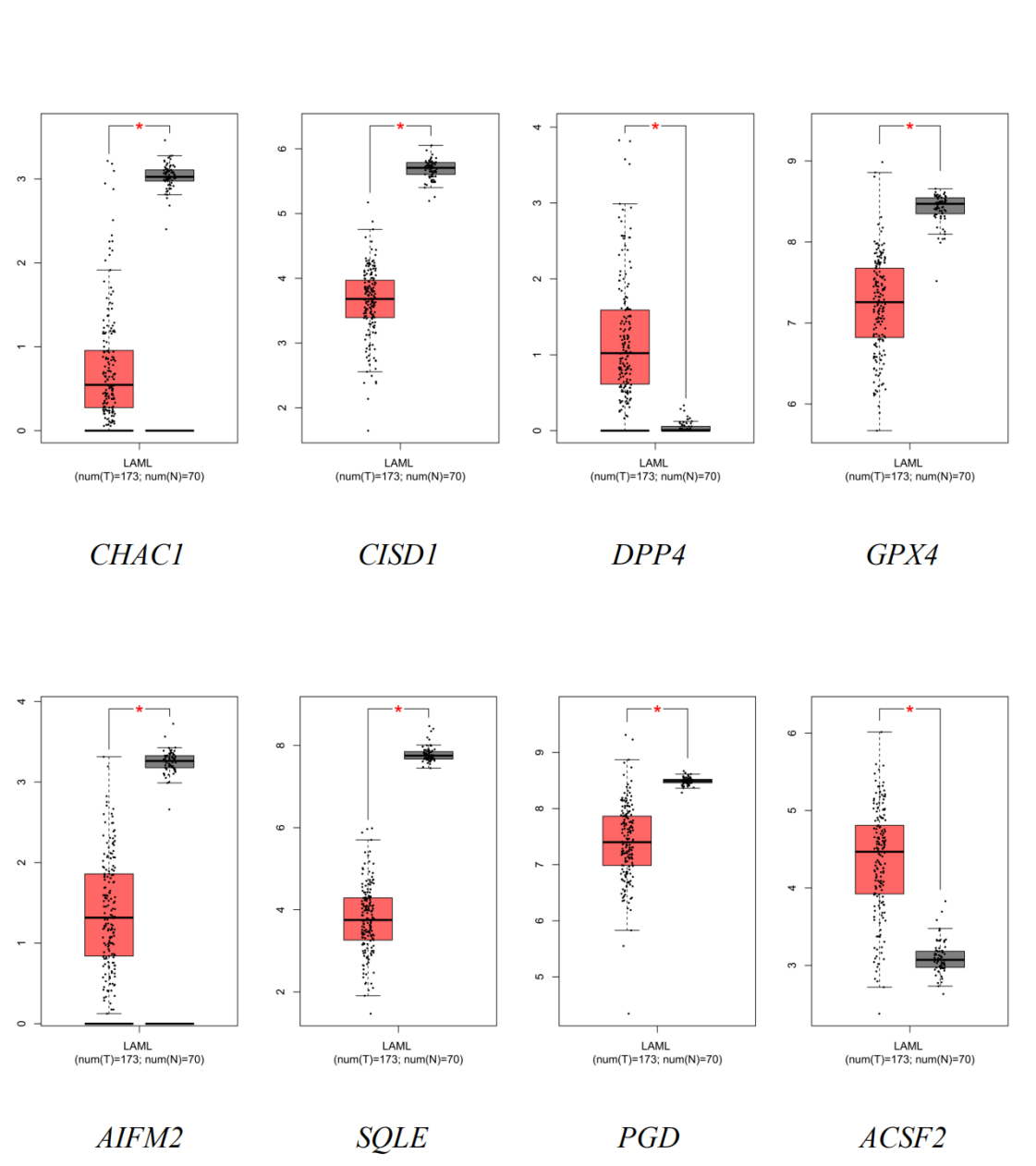

Supplement: Supplementary file 4 [file Image1.JPEG]
